# Effect of surface electrode recording area on compound muscle action potential scan processing for motor unit number estimation

**DOI:** 10.3389/fnins.2024.1382871

**Published:** 2024-05-22

**Authors:** Dan Zhang, Zhiyuan Lu, Weijun Gong, Ping Zhou

**Affiliations:** ^1^School of Rehabilitation Medicine, Shandong Second Medical University, Weifang, Shandong, China; ^2^School of Rehabilitation Science and Engineering, University of Health and Rehabilitation Sciences, Qingdao, Shandong, China; ^3^Department of Neurological Rehabilitation, Beijing Rehabilitation Hospital, Capital Medical University, Beijing, China

**Keywords:** MScanFit, motor unit number estimation, compound muscle action potential scan, electromyography, electrode recording area

## Abstract

**Introduction:**

MScanFit is a model-based algorithm for motor unit number estimation (MUNE) from compound muscle action potential (CMAP) scan data. It is a clinically applicable tool because of its quick and automatic implementation. Electrodes with different recording areas were employed to record CMAP scan data in existing studies. However, the effect of electrode recording area on MScanFit MUNE and other CMAP scan parameters has not been studied.

**Methods:**

CMAP scan was performed on the abductor pollicis brevis muscle of both hands on 14 healthy subjects using three different electrodes with recording areas of 10 mm × 10 mm, 11 mm × 14 mm, and 22 mm × 26 mm, respectively. Motor unit number was estimated using MScanFit for each CMAP scan. Two motor unit number index parameters, i.e., D50 and step index (STEPIX), were also derived from the CMAP scan data.

**Results:**

No significant difference in D50, STEPIX, and MScanFit MUNE was observed across three different electrode recording areas, although the amplitude of CMAP decreased significantly when a larger electrode was used. Intraclass correlation coefficients of 0.792 and 0.782 were obtained for MScanFit MUNE and STEPIX, respectively.

**Discussion:**

Compared with CMAP amplitude, D50, STEPIX, and MScanFit MUNE are less sensitive to variation in electrode recording area. However, the repeatability of MScanFit MUNE could be compromised by the inconsistency in the electrode recording area.

## 1 Introduction

The reduction of motor units may lead to muscle weakness and muscle atrophy in neuromuscular diseases. Motor unit number estimation (MUNE) is a powerful tool for tracking loss of motor units and the compensatory phenomenon of collateral reinnervation (Gooch et al., 2014). A variety of MUNE methods have been proposed in past decades, but those traditional MUNE methods such as incremental stimulation MUNE and multiple point stimulation MUNE (de Carvalho et al., 2018; Wright et al., 2021) are likely biased to the sampling of motor units. By contrast, compound muscle action potential (CMAP) scan aims to gradually activate/deactivate all motor units by applying hundreds of transcutaneous stimuli to the motor nerve across a wide range of intensities (Blok et al., 2007). Compared with traditional MUNE methods, CMAP scan is less biased to the sampling of motor units, and various methods have been developed to process CMAP scan for examination of neuromuscular disorders (Sleutjes et al., 2014; Nandedkar et al., 2022; Chen et al., 2023a,b; Lu et al., 2023b). Of particular note, Bostock proposed a model-based MUNE algorithm named MScanFit, which estimates the number of motor units by fitting the detailed stimulus–response curve recorded from a CMAP scan (Bostock, 2016). MScanFit possesses the advantage of automated and quick (typically taking only a few minutes) implementation, making it so far the most often used CMAP scan processing method in basic and clinical electrophysiological studies (Kristensen et al., 2019; Zong et al., 2021; Schneider et al., 2023).

CMAP scan curve can be affected by experimental parameters including the number and the width of electrical stimuli. For example, our previous studies show that CMAP scan curve becomes denser when the number of stimuli is increased, which leads to an increase in derived step index (STEPIX; Lu et al., 2023a). Although MScanFit is not sensitive to the number of stimuli, it is significantly affected by the width of stimuli (Zong et al., 2020).

Electrode recording area (the area of the recording surface of an electrode) is another major factor affecting CMAP, especially on its shape and amplitude due to the different filtering effects (Wee and Ashley, 1990; Chang et al., 1993; Jonas et al., 1999; Barkhaus et al., 2006). The electrode recording area used for CMAP scan studies covered a wide range in literature from greater than 400 mm^2^ (Sørensen et al., 2022, 2023) to around 100 mm^2^ (Li et al., 2018; Song et al., 2023). Furthermore, inconsistent electrode recording areas were reported when examining the same muscle (Sleutjes et al., 2021). For example, when performing CMAP scan recordings on the abductor pollicis brevis (APB) muscle, square electrodes with the size of 30 mm × 22 mm (with the recording area of 474 mm^2^) and 30 mm × 24 mm (detailed recording area was not reported) were used in Araújo et al. (2015) and Sørensen et al. (2023), respectively, while disk electrodes with the diameter of 10 mm (i.e., 79 mm^2^) and 13 mm (i.e., 133 mm^2^) were applied in Stikvoort García et al. (2022) and Song et al. (2023), respectively. Note that 2-dimensional electrode arrays up to 128 channels have also been used for MUNE, where electrodes with diameters smaller than 2 mm are commonly used (Sleutjes et al., 2016).

The effect of electrode recording area on CMAP implies that the CMAP scan curves could also vary with different surface electrodes. However, it still remains unclear how the different surface electrode recording areas may affect CMAP scan processing parameters, such as MScanFit MUNE. The objective of this study was, therefore, to assess the effect of electrode recording area on MScanFit MUNE and other CMAP scan parameters. In addition, the repeatability of these parameters using different electrode recording areas was quantified.

## 2 Methods

### 2.1 Experimental protocol

Three electrodes with different recording areas were used for CMAP scan recording in this study. Their recording areas are 10 mm × 10 mm (denoted as E1), 11 mm × 14 mm (denoted as E2), and 22 mm × 26 mm (denoted as E3), respectively.

Fourteen right-handed healthy subjects (7 males and 7 females, aged 30.6 ± 9.5 years) participated in this study. Each subject’s bilateral APB muscles were recorded. The order of the left and right hand was randomized. In the experiment, each subject was seated comfortably in a chair with his/her testing hand rested on a table and restrained in the pronation position. CMAP scan was performed three times on each hand using one of the three active electrodes in a random order, while all the other experimental parameters remained the same. Before each recording, the subject was given sufficient rest to avoid mental and muscle fatigue.

### 2.2 CMAP scan recording

Before each recording, the range of stimulating current intensity was determined by performing an automatic search. The range of current intensity was then manually tuned in order to cover the entire motor unit recruitment range. The pulse duration was set to 0.1 ms, the number of stimuli was set to 500, and the frequency of stimuli was set to 2 Hz. All the data were collected using Nicolet EDX system (Natus Neurology Incorporated, Middleton, WI, United States).

**Figure 1:**
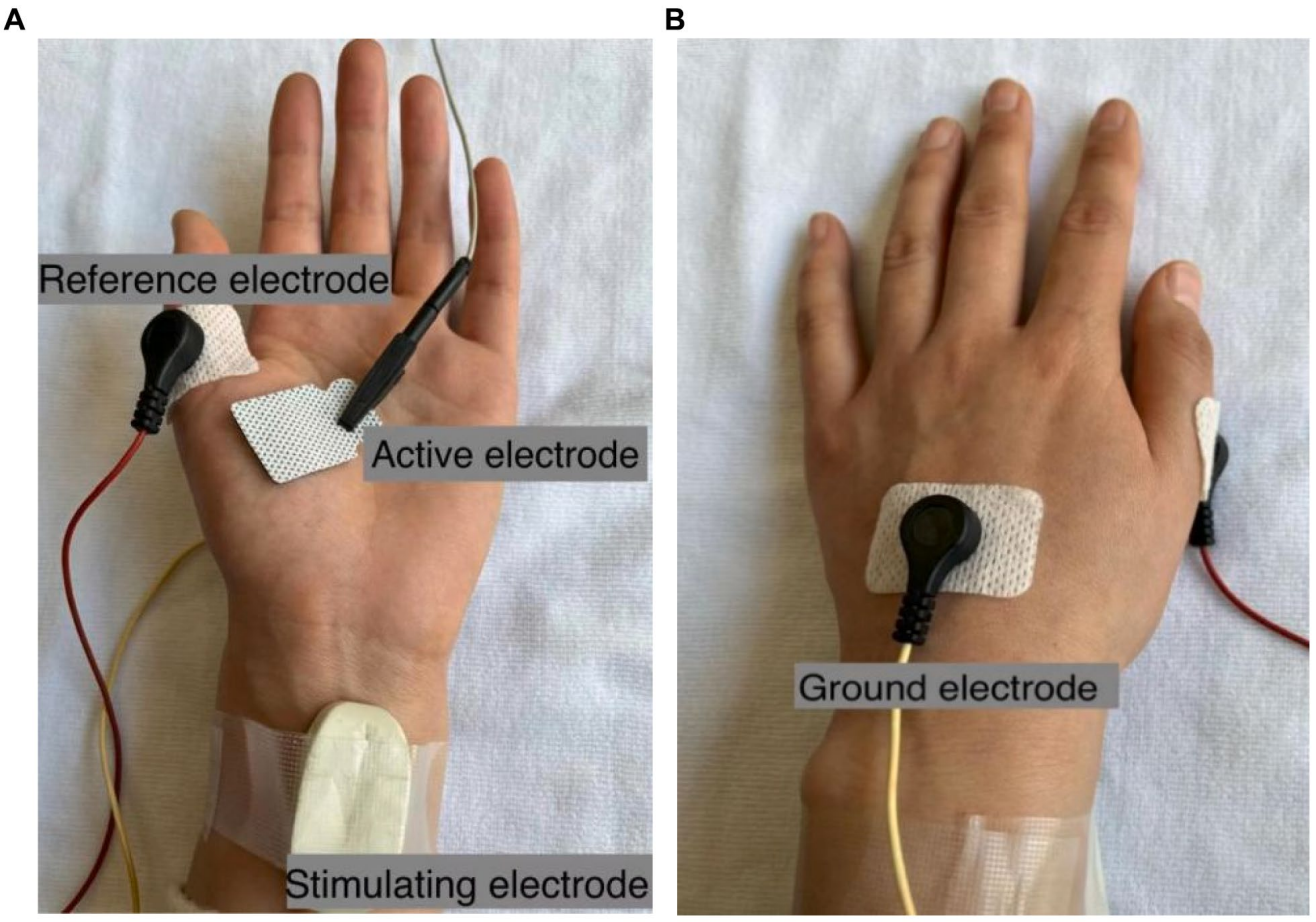
Electrode configurations for CMAP scan recording of the APB muscle. The active electrode E3 with the size of 22 mm × 26 mm is shown as an example in **(A)**, and the ground electrode is shown in **(B)**.

As shown in Figure 1, the active electrode was placed on the abdominal eminence of the APB muscle, and the reference electrode was placed on the metacarpophalangeal joint of the thumb. The ground electrode was placed on the bony protuberance on the back of the hand between the active electrode and the reference electrode. The stimulating electrode (Ag/AgCl electrode) with two contact surfaces spaced 20 mm apart and each having a diameter of 9 mm, was placed 1–2 cm proximal to the wrist to activate the median nerve. The electrode was coated with conductive paste and the cathode was oriented distally. Both recording and stimulating electrodes were carefully tuned in order to optimize electrode positions where the largest CMAP amplitude can be evoked with a relatively low stimulating current intensity. Once the stimulating site was determined, the electrode was fixed with surgical tape or self-adherent wrap. Alcohol pads were used to clean the thumb, thenar, wrist, and back of the hand before the electrodes were attached.

### 2.3 CMAP scan data analysis

The MScanFit program (free version 2016; Bostock, 2016) was applied to estimate motor unit number. Each recording was analyzed multiple times until three valid estimations were obtained (i.e., percentage error < 7%), and the one with the smallest error was accepted. The pre-scan and post-scan limits were manually selected each time, while all the other settings remained at their default values.

D50, step index, S0, S100, and the difference between S0 and S100 (i.e., S100 − S0) were also derived from each scan using a customized Matlab script. D50 is the number of largest consecutive differences from each scan that are required to build up 50% of the maximum CMAP amplitude (Sleutjes et al., 2014). STEPIX, which is a recently proposed index based on the logarithmic relation between the step amplitude and step number in a CMAP scan, reflects the number of motor units (Nandedkar et al., 2022). S0 is the maximum electrical intensity that cannot activate any motor unit. S100 is the minimum electrical intensity that can activate all the motor units.

### 2.4 Statistical analysis

One-way repeated measures analysis of variance was performed to examine the differences in the maximum CMAP amplitude, S0, S100, S100 − S0, D50, STEPIX, and MScanFit MUNE parameters for different electrode recording areas. Statistical significance was set as *p* < 0.05. Repeatability was quantified using consistency intraclass correlation coefficient (ICC) if significant difference was observed; otherwise, absolute agreement ICC was used. Results are presented as mean ± standard error.

## 3 Results

Mild or tolerable pain was reported by the subjects in our experiment. The CMAP amplitude of one subject’s left hand was lower than 50% of the amplitude of his right hand, and thus CMAP scan data from his left hand was excluded. As a result, a total of 81 CMAP scan curves (3 trials per hand × 27 hands) recorded from 14 subjects were analyzed.

**Figure 2:**
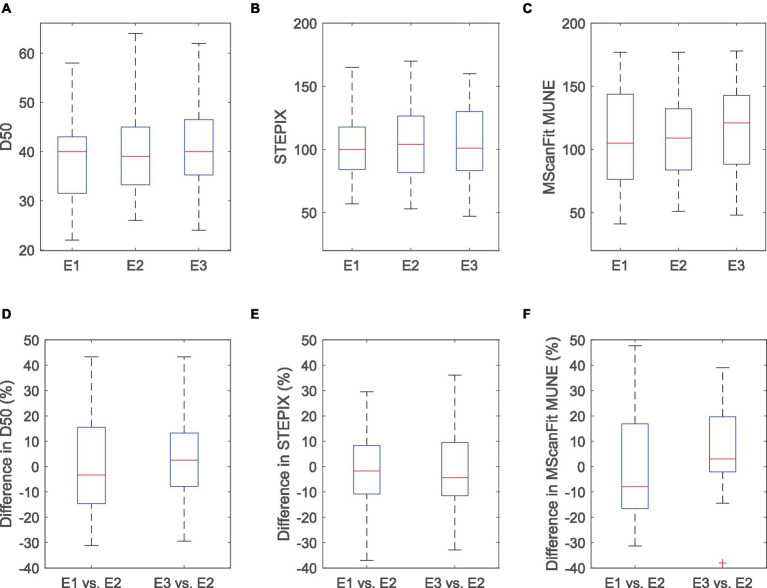
D50 **(A)**, STEPIX **(B)**, MScanFit MUNE **(C)**, and the difference in D50 **(D)**, STEPIX **(E)**, and MScanFit MUNE **(F)** between CMAP scan curves of the APB muscle of healthy subjects recorded using electrodes with different recording areas. The difference was calculated as (Parameter derived from E1 or E3−Parameter derived from E2)/Parameter derived from E2 for each subject. One outlier (above 50% in both STEPIX and MScanFit) is not shown in this figure.

The maximum CMAP amplitude of the APB muscle was 10.18 ± 0.58 mV for electrode E1, 9.65 ± 0.56 mV for electrode E2, and 8.54 ± 0.50 mV for electrode E3 (as shown in Table 1). Significant difference was observed across the three electrodes (*p* < 0.0001), and the maximum CMAP amplitude decreased at a larger electrode recording area. No significant difference was observed across the three electrode recording areas in S0 (*p* = 0.592), S100 (*p* = 0.482), S100 − S0 (*p* = 0.536), D50 (*p* = 0.463), STEPIX (*p* = 0.654), or MScanFit MUNE (*p* = 0.155). The ICC of MScanFit MUNE (0.792) was greater than that of the other two indexes (0.782 for STEPIX and 0.686 for D50). The median value of difference in D50, STEPIX, and MScanFit MUNE between individual subject’s CMAP scan curves recorded using electrodes with different recording areas was distributed close to 0 (i.e., no significant difference across three electrodes). However, it was observed that the difference in these parameters between two electrodes could be distributed in a large range, as shown in Figure 2.

**Table 1:** Parameters derived from CMAP scan of the APB muscle on healthy subjects using electrodes with different recording areas.

	10 mm × 10 mm	11 mm × 14 mm	22 mm × 26 mm	Significance	ICC^*^
Maximum CMAP (mV)	10.18 ± 0.58	9.65 ± 0.56	8.54 ± 0.50	*p* < 0.0001	0.934
S0 (mA)	10.01 ± 0.51	10.06 ± 0.56	10.23 ± 0.54	*p* = 0.592	0.914
S100 (mA)	19.21 ± 0.88	19.00 ± 0.80	19.53 ± 0.88	*p* = 0.482	0.867
S100 − S0 (mA)	9.19 ± 0.58	8.95 ± 0.51	9.31 ± 0.58	*p* = 0.536	0.831
D50	38.85 ± 1.64	39.37 ± 1.64	40.44 ± 1.63	*p* = 0.463	0.686
STEPIX	103.67 ± 5.50	106.59 ± 6.00	103.37 ± 5.81	*p* = 0.654	0.782
MScanFit MUNE	107.37 ± 7.33	109.07 ± 6.57	115.52 ± 6.75	*p* = 0.155	0.792

^*^All these ICC values are the absolute agreement type, except the ICC of the maximum CMAP, which is the consistency type.

**Figure 3:**
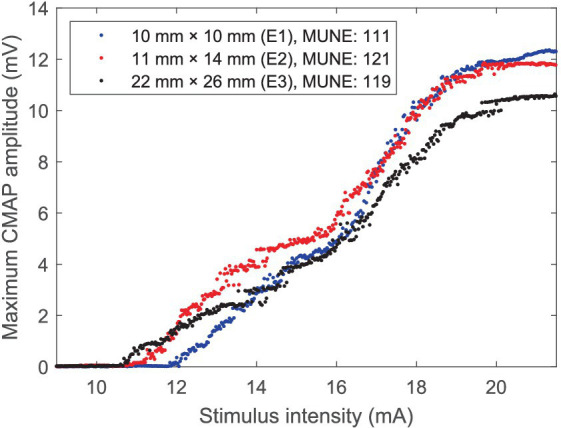
CMAP scan curves of the APB muscle of a representative healthy subject recorded using three electrodes with different recording areas.

Three CMAP scan curves recorded using electrode E1, E2, and E3 from the APB muscle of a representative subject are demonstrated in Figure 3. It is worth noting that the curves recorded using electrode E2 and E3 demonstrate a close pattern, and there is only a difference of 2 (or 1.7%) in MScanFit MUNE although the difference in maximum CMAP amplitude is as high as 1.13 mV (or 9.5%).

## 4 Discussion

This study presents a novel analysis of CMAP scan parameters with respect to the different electrode recording areas. By testing three different electrodes on CMAP scan recordings of the APB muscle we observed that changes in the electrode recording area had no significant impact on the examined CMAP processing parameters including D50, STEPIX and MScanFit MUNE. In recent years, different electrodes or recording areas have been used in CMAP scan experiments on various muscles. For example, a disk electrode with 11 mm in diameter was used to examine the abductor hallucis muscle (Li et al., 2018); a smaller disk electrode with 10 mm in diameter was used to collect CMAP scan data from the first dorsal interosseous (FDI) muscle (Zong et al., 2020). Additionally, some researchers used 13 mm diameter disk electrodes to examine the FDI and abductor digiti minimi (ADM) muscles (Song et al., 2023). For the APB muscle examined in this study, literature shows that the surface electrode used for CMAP scan recording ranged from 10 mm to 15 mm in diameter (i.e., 79 mm^2^ to 177 mm^2^; Farschtschi et al., 2017; Stikvoort García et al., 2022; Zong et al., 2022c; Song et al., 2023). Square electrodes with different recording areas were also reported for APB muscle CMAP scan recordings (Araújo et al., 2015; Sørensen et al., 2023). Considering various recording areas reported in previous CMAP scan studies, we chose to use three electrodes with recording areas of 100 mm^2^, 154 mm^2^, and 572 mm^2^, respectively. The three electrode recording areas used in this study can cover the commonly used surface electrodes in research and clinical practice.

In addition to motor unit number parameters (e.g., MScanFit MUNE), it was also observed that the three different electrode recording areas had no significant impact on CMAP scan’s stimulus intensity parameters including S0, S100, and S100 − S0. This is not surprising since the stimulus intensities that elicit motor unit responses are independent of the electrode size. Our results indicate that both large and small electrode recording areas have a similar sensitivity in capturing the recruited motor unit activity of the examined muscle.

The only observed significant difference across three different electrode recording areas was CMAP amplitude. Our results indicated that increased electrode recording area significantly reduced CMAP amplitude of the APB muscle. This is consistent to previous experimental (Wee and Ashley, 1990; Barkhaus et al., 2006) and theoretical findings (Fuglevand et al., 1992). The same tendency was also reported by Chang et al. (1993) although the significance level was not reached. Large surface electrodes can capture relatively more muscle volume but impose an increased low pass filtering effect on the recorded signal compared with small ones. These two factors have opposite effects on the CMAP amplitude. For the examined APB muscle in this study, it seems the difference in low pass filtering effect caused by different surface electrodes was more dominant than the difference in captured muscle volume, thus the CMAP amplitude was reduced with increased recording surface area. Nonetheless, the alterations in CMAP amplitude did not have a significant influence on its processing parameters, such as MScanFit MUNE. This is likely because MScanFit MUNE applies a number of operations to refine the CMAP scan model to meet the predefined error score, including adjusting individual motor unit parameters, splitting or merging motor units, etc. This is different from conventional MUNE methods, calculated as the ratio of the CMAP measurement to the mean motor unit action potential measurement estimated from a small sample of motor units.

The repeatability of CMAP scan parameters was also examined in this study across three different surface electrodes. The repeatability of MScanFit MUNE across the three different electrodes was observed to be slightly higher than that of the two index parameters (i.e., D50 and STEPIX). The repeatability of MScanFit MUNE can be exemplified by Figure 3, as it indicates a relative large variation in CMAP scan amplitude (induced from different electrodes) does not necessarily impose a similar extent of variation in MScanFit MUNE. Existing studies reported that the test–retest repeatability of MScanFit MUNE was excellent using the same electrode for CMAP scan recordings. For example, the ICC of test–retest repeatability achieved 0.93, 0.90, and 0.96 in our previous studies on the abductor hallucis (Li et al., 2018), the anconeus (Zong et al., 2022b), and the second lumbrical (Zong et al., 2022a) muscles, respectively. ICCs greater than 0.8 were also reported for MScanFit MUNE in three repeated tests on the APB, FDI, and ADM muscles (Higashihara et al., 2020). In this study, as expected, although MScanFit MUNE values were not significantly different across three different surface electrode recording areas, the repeatability of MScanFit MUNE was not as high as previously reported numbers, due to inconsistency in surface electrode recording area.

As an important neuromuscular electrophysiological method, MUNE is often used to compare the difference between two groups (for example, between healthy control subjects and subjects with neuromuscular diseases) in a cross-sectional study or track the same muscle in a longitudinal study. Although group analysis revealed no significant difference in MScanFit MUNE of the APB muscle across three different surface electrodes, variation up to ±50% in individual subject was observed between two different electrode recording areas. Such a variation may reduce the reliability of tracking motor unit loss. Therefore, we advocate the same experimental settings (including the same recording electrode) should be used in MUNE studies for both research and clinical settings. This can help to avoid confounding factors for comparing MUNE and other CMAP scan parameters in different situations.

The current study is limited by only examining the APB muscle of neurologically intact subjects. It remains to be determined whether the findings can be generalized to other muscles, particularly to those large muscles. In addition, it is important in the future work to investigate how different electrode recording areas may affect the sensitivity of MScanFit and other CMAP scan processing parameters in quantifying motor unit number and size changes in clinical application.

## 5 Conclusion

The effect of electrode recording area on MScanFit MUNE and other parameters derived from a CMAP scan was assessed by testing three different electrode recording areas. The experimental results from APB muscles indicate that although CMAP amplitude was sensitive to surface electrode recording area, CMAP scan processing parameters including D50, STEPIX, and MScanFit MUNE were not significantly affected by the changes in electrode recording area. However, inconsistency in electrode recording area may compromise the repeatability of CMAP scan processing. The findings of the study can help to understand the effect of experimental factors on different CMAP scan parameters, thus facilitating their analysis and interpretation.

## Data availability statement

The raw data supporting the conclusions of this article will be made available by the authors, without undue reservation.

## Ethics statement

The studies involving humans were approved by the Ethics Committee of the University of Health and Rehabilitation Sciences. The studies were conducted in accordance with the local legislation and institutional requirements. Written informed consent for participation in this study was provided by the participants.

## Author contributions

DZ: Data curation, Formal analysis, Investigation, Software, Visualization, Writing – original draft. ZL: Conceptualization, Funding acquisition, Investigation, Methodology, Project administration, Resources, Software, Validation, Writing – original draft. WG: Methodology, Project administration, Validation, Resources, Supervision, Writing – review & editing. PZ: Conceptualization, Funding acquisition, Methodology, Resources, Supervision, Validation, Writing – review & editing.
